# microRNA-mRNA Profile of Skeletal Muscle Differentiation and Relevance to Congenital Myotonic Dystrophy

**DOI:** 10.3390/ijms22052692

**Published:** 2021-03-07

**Authors:** Sarah U. Morton, Christopher R. Sefton, Huanqing Zhang, Manhong Dai, David L. Turner, Michael D. Uhler, Pankaj B. Agrawal

**Affiliations:** 1 Division of Newborn Medicine, Boston Children’s Hospital, Boston, MA 02115, USA; 2 Department of Pediatrics, Harvard Medical School, Boston, MA 02115, USA; 3 School of Medicine, University of North Carolina, Chapel Hill, NC 27516, USA; christopher_sefton@med.unc.edu; 4 Michigan Neuroscience Institute, University of Michigan, Ann Arbor, MI 48109, USA; hqzh@med.umich.edu (H.Z.); daimh@umich.edu (M.D.); dlturner@med.umich.edu (D.L.T.); muhler@med.umich.edu (M.D.U.); 5 Department of Biological Chemistry, University of Michigan, Ann Arbor, MI 48109, USA; 6 Division of Genetics and Genomics, Boston Children’s Hospital, Boston, MA 02115, USA; 7 The Manton Center for Orphan Disease Research, Boston Children’s Hospital, Boston, MA 02115, USA

**Keywords:** microRNA, muscle differentiation, congenital myotonic dystrophy

## Abstract

microRNAs (miRNAs) regulate messenger RNA (mRNA) abundance and translation during key developmental processes including muscle differentiation. Assessment of miRNA targets can provide insight into muscle biology and gene expression profiles altered by disease. mRNA and miRNA libraries were generated from C2C12 myoblasts during differentiation, and predicted miRNA targets were identified based on presence of miRNA binding sites and reciprocal expression. Seventeen miRNAs were differentially expressed at all time intervals (comparing days 0, 2, and 5) of differentiation. mRNA targets of differentially expressed miRNAs were enriched for functions related to calcium signaling and sarcomere formation. To evaluate this relationship in a disease state, we evaluated the miRNAs differentially expressed in human congenital myotonic dystrophy (CMD) myoblasts and compared with normal control. Seventy-four miRNAs were differentially expressed during healthy human myocyte maturation, of which only 12 were also up- or downregulated in CMD patient cells. The 62 miRNAs that were only differentially expressed in healthy cells were compared with differentiating C2C12 cells. Eighteen of the 62 were conserved in mouse and up- or down-regulated during mouse myoblast differentiation, and their C2C12 targets were enriched for functions related to muscle differentiation and contraction.

## 1. Introduction

MicroRNAs are 21- to 22-nucleotide RNAs that function in post-transcriptional regulation of gene expression [1]. miRNAs modulate gene expression by binding to 3′ untranslated regions (UTRs) of mRNA and recruiting protein complexes that modulate target mRNA abundance or translational efficiency [2]. Their effects on protein expression have been implicated in the regulation of a wide range of biological functions and diseases [3,4,5]. Several miRNAs have been demonstrated to regulate muscle differentiation [6,7]. MyomiRs, including, miR-1, miR-133a, miR-206, miR-208a/b, and miR-499, are important for muscle development [8]. For example, mice lacking miR-206 develop a muscular dystrophy phenotype with decreased bulk, muscle fibrosis, and fatty infiltrate [9]. miR-208a/b and miR-499 are necessary for muscle fiber type determination: overexpression of these miRNAs causes total conversion to slow twitch fibers while depletion leads to loss of slow twitch fibers [10]. Multiple miRNAs may function coordinately to target multiple mRNAs within a molecular pathway, or a single miRNA may target multiple mRNAs within a pathway: miR-1, miR-133a and miR-206 have been shown to regulate cell cycle progression and gene transcription through targeting of *Hdac4*, *Pax7*, *Sp1* and *Cyclin D* [6,11,12,13]. Additionally, miR-133a has been shown to target *Fgfr1* and *Pp2ac*, which both contribute to ERK1/2 signaling, downregulating that pathway to promote differentiation and inhibit proliferation [14]. Furthermore, miRNA dysregulation has been implicated in muscular disorders including Duchene muscular dystrophy [15,16], myotonic dystrophy (CMD) [17,18], and Becker muscular dystrophy [15].

Pairing of miRNA and mRNA expression data allows for prediction of miRNA targets based on reciprocal expression, strengthening predictions made from presence of a potential miRNA binding site alone. In this study, we analyze mRNA and miRNA expression data during C2C12 myoblast differentiation to identify candidate miRNA-mRNA regulatory pairs and assess for functional enrichment of predicted target mRNAs. Differential miRNA expression during muscle differentiation was also determined using published data from CMD and healthy myoblasts, and unique miRNA expression patterns were identified and compared with C2C12 cells. This analysis highlights importance of miRNAs during normal muscle development, and identifies potential regulatory functions during muscle differentiation.

## 2. Results

### 2.1. Differential Expression of mRNA and miRNA during Myoblast Differentiation

**Figure 1:**
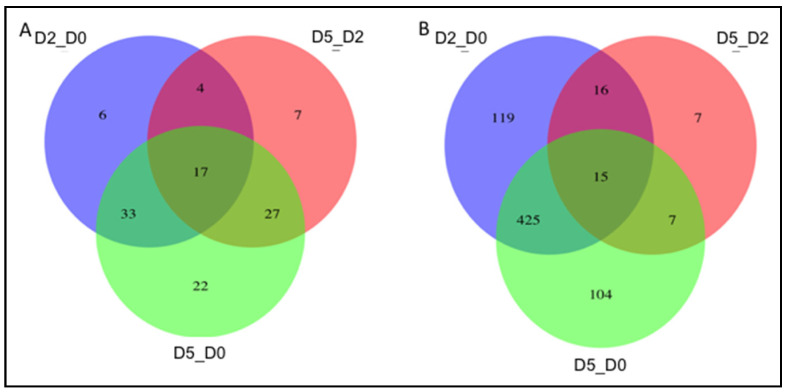
Differentially expressed (**A**) miRNA and (**B**) mRNA at day 2 vs. day 0 (D2_D0, myoblast to early myocyte), day 5 vs. day 0 (D5_D0, myoblast to mature myocyte), and day 5 vs. day 2 (D5_D2, early myocyte to mature myocyte).

mRNA and miRNA levels were measured at three different time points: day 0 (D0, myoblast), day 2 (D2, early myocyte) and day 5 (D5, mature myocyte). Differential mRNA and miRNA expression were determined pairwise across these three timepoints: day 2 vs. day 0 (D2_D0), day 5 vs. day 0 (D5_D0) and day 5 vs. day 2 (D5_D2). On evaluating the above three time-interval comparisons, 112 miRNA and 693 mRNA were differentially expressed in at least one of them (Figure 1, Supplemental Table S1). D5_D0 had the largest number of differentially expressed miRNAs (99, 100%), and the majority (77, 77%) of those miRNAs were also differentially expressed in at least one other comparison. By contrast, there were a similar number of mRNAs differentially expressed during both the D2_D0 and D5_D0 comparisons (675 and 551), and the majority of those mRNAs were shared between those two comparisons (440, 80% of D5_D0). The number of miRNAs or mRNAs that were differentially expressed at both D2_D0 and D5_D2 (21/60 miRNAs and 31/575 mRNAs shared at D2_D0 compared to 21/56 miRNAs and 31/45 mRNAs shared at D5_D2) was not more than would be expected by chance (hypergeometric *p*-value 1.0 for both miRNA and mRNA).

### 2.2. Pathway Enrichment for Differentially Expressed mRNAs

**Figure 2:**
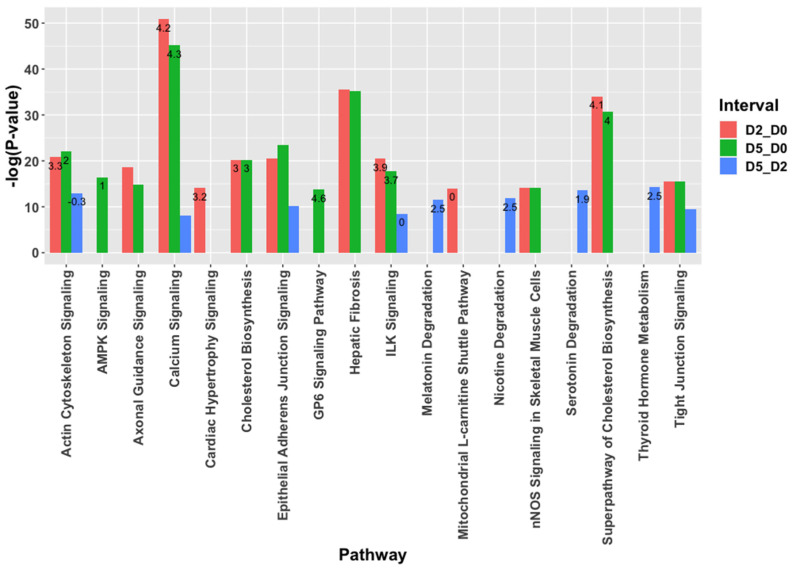
Top functional enrichments among differentially expressed mRNAs during muscle differentiation. Only the most significantly enriched term among closely related terms was included. Z-score for pathway activity is written on the relevant bar where applicable.

The differentially expressed mRNAs were analyzed for functional enrichment in biological pathways (Figure 2). Overall, the pathway changes present on D2 vs. D0 were also seen on D5 vs. D0 comparison. Calcium signaling was upregulated with the greatest significance and positive z-score in both D2 and D5 compared to D0. Actin cytoskeleton signaling and cholesterol synthesis pathways also had positive z-scores in D2_D0 and D5_D0 comparisons. In contrast, the actin cytoskeleton pathway was downregulated on D5 vs. D2 comparison. Meanwhile, mitochondrial L-carnitine shuttle was uniquely enriched on D2 vs. D0 comparison while AMPK and G6P signaling pathways were upregulated on D5 vs. D0 comparison. Pathways significantly upregulated on D5_D2 comparison were: metabolism of thyroid hormone, nicotine, melatonin, and serotonin.

### 2.3. Pathway Enrichment for Predicted mRNA Targets of Differentially Expressed miRNAs

Predicted mRNA-miRNA interactions during muscle differentiation were identified (Supplemental Table S2) and then predicted target mRNAs were analyzed for enrichment in biological pathways (Table 1). Similar to enrichment among all differentially expressed mRNAs, calcium signaling was the most significantly upregulated pathway in the D2 vs. D0 and D5 vs. D0 comparisons while pathways related to degradation of nicotine, melatonin, and serotonin were all upregulated in D5 compared to D2. By contrast, there were several functional enrichments only observed among predicted mRNA targets including PPAR, protein kinase A, and RhoA signaling in the D5_D0 comparison. Further, D2_D0 targeted mRNAs were enriched for pathways predicted to be inhibited during this early interval: metabolism of thyroid hormone, nicotine, and melatonin. Those same pathways had been predicted to be activated among all differentially expressed mRNAs at D5_D2. Cyclins and cell regulation pathways were downregulated in both the D2_D0 and D5_D0 comparisons, while G1/S checkpoint and S-phase entry pathways were upregulated in D2_D0. In contrast to the total differential expression mRNA analysis, cholesterol synthesis pathways were not enriched among mRNAs targeted by miRNAs in the D2_D0 and D5_D0 comparisons.

### 2.4. miRNAs in Calcium Signaling

**Figure 3:**
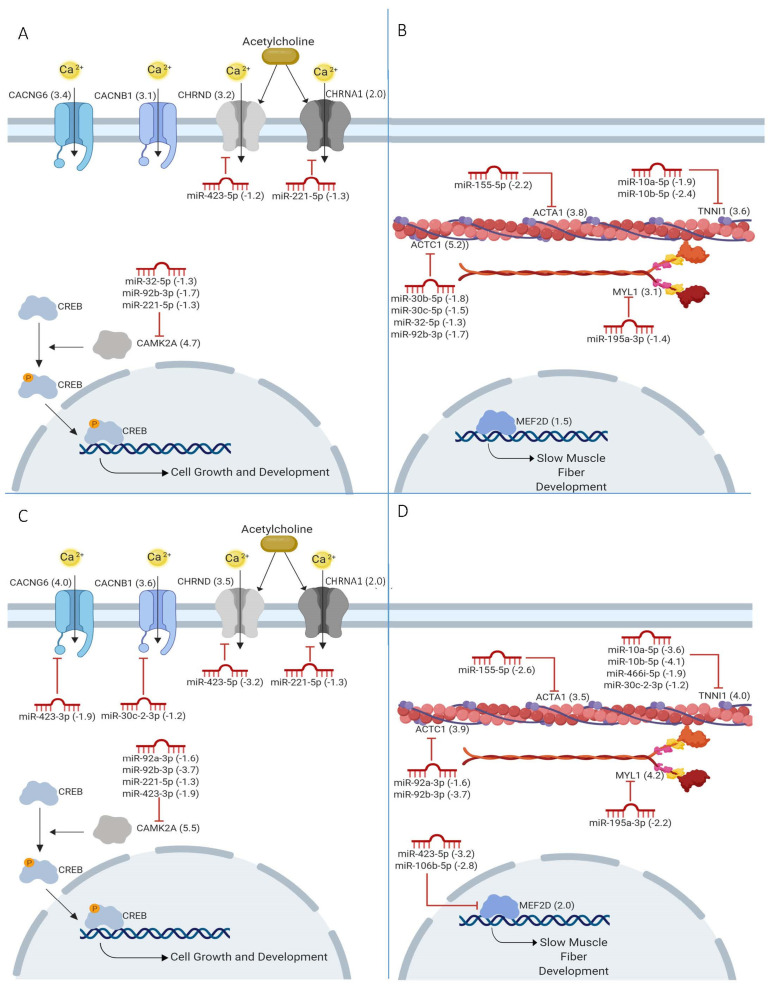
miRNAs are predicted to target multiple mRNAs at D2_D0 (**A**,**B**) and D5_D0 (**C**,**D**) that encode proteins related to calcium signaling (**A**,**C**) and sarcomeres (**B**,**D**).

Redundancy in miRNAs and targeted mRNAs was explored in the calcium signaling pathway at the D2_D0 and D5_D0 comparisons (Figure 3A,C). Of genes predicted to be targeted by miRNA, *Camk2a* was the only gene with multiple predicted miRNA interactions (miR-32-5p, miR-92b-3p, and miR-221-5p in D2_D0; miR-92a-3p, miR-92b-3p, miR-221-5p and miR-423-3p in D5_D0). Reciprocally, two miRNAs (miR-221-5p in D2_D0; miR-423-3p and miR-221-5p in D5_D0) have multiple mRNA targets within the calcium signaling pathway. In addition to the calcium pathway genes annotated by Ingenuity Pathway Analysis (IPA), 2 predicted targets of miR-423-5p, *Lynx1* and *Pvalb*, have also been implicated in calcium signaling through stabilization of acetylcholine receptors and calcium binding, respectively [19,20]. *Lynx1* was also a predicted target of miR-423-3p and miR-221-5p, and both of those miRNAs were predicted to target two subunits of the muscle acetylcholine receptor (*Chrnd1* and *Chrna1*, respectively) [21,22].

### 2.5. Sarcomere Genes Targeted by miRNAs

Sarcomere structures, such as actin, troponin, and myosin, were also predicted to be targeted by several miRNAs during myoblast differentiation (Figure 3B,D). Both *Actc1* and *Tnni1* were the predicted targets of multiple miRNA (miR-30b/c-5p, miR-32-5p, and miR-92a/b-3p; and miR-10a-5p, miR-10b-5p, miR-466i-5p, and miR-30c-2-3p, respectively). Additionally, *Mef2d*, a member of the MEF2 family of transcription factors known to regulate the differentiation of myoblasts and maintenance of sarcomeres [23,24], was predicted to be targeted by miR-423-5p and miR-106b-5p. Several miRNAs were predicted to target genes related to both calcium signaling and sarcomeres structures including miR-30c-2-3p, miR-32-5p, miR-92a-3p, miR-92b-3p, and miR-423-5p.

### 2.6. myomiR Target Functions

**Figure 4:**
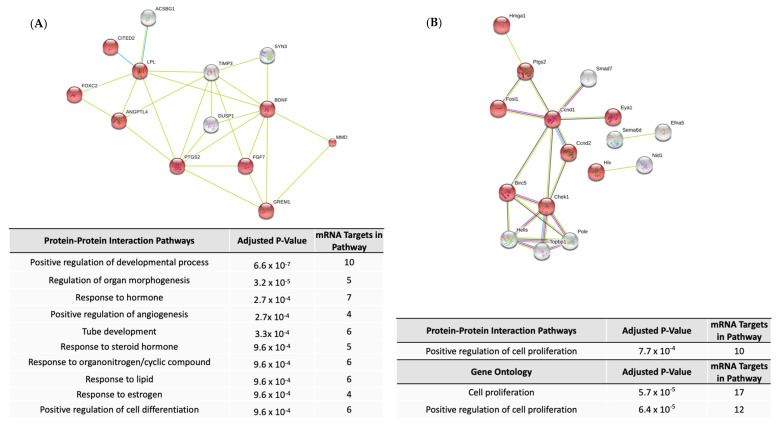
Functional enrichment among predicted mRNA targets of myomiRs (**A**) and miR-503 cluster miRNAs (**B**) based on protein-protein interaction and gene ontology analysis. In A, red denotes proteins that contribute to positive regulation of developmental process. In B, red spheres denotes proteins that are involved in proliferation. White spheres denote proteins that lack a directional pattern of effect. Only the most significantly enriched term among closely related terms was included.

Eight miRNAs (miR-1, miR-133a, miR-133b, miR-206, miR-208a, miR-208b, miR-499a, and miR-499b) are important for muscle development and are known as myoMirs. Of note, all myomiRs were significantly upregulated during muscle differentiation except miR-208a and miR-208b (Supplemental Table S1). Differentially expressed myoMirs were predicted to target mRNA with functions related to regulation of developmental processes, regulation of structural and organ morphogenesis, programmed cell death and apoptosis, and response to endogenous stimulus and hormones by gene ontology analysis and protein-protein interaction analysis (Figure 4A, Supplemental Table S3).

### 2.7. Loss of miRNA Differential Expression in CMD

Seventy-four miRNAs were significantly up- or downregulated during control human myoblast differentiation from myoblasts to mature myocytes (day 0 to day 3). Sixty-two of those 74 miRNAs were differentially expressed in myoblasts derived from patient with a moderate (DM13) CMD genotype. However, only 12 of the 74 were up- or downregulated in cells from the patient with severe CMD (DM15). All of those 12 were also differentially expressed in DM13 cells (Supplemental Table S4). Five of the 12 miRNAs with preserved differential expression in DM15 cells were known myomiRs (miR-1, miR-133a-3p, miR-133a-5p, miR-133b, miR-206). Among the 62 miRNAs with loss of significant up- or downregulation during DM15 myoblast differentiation, 25 miRNAs were conserved between human and mouse. Of those, 18 were also up- or downregulated during C2C12 differentiation (2 at D2_D0 only, 4 at D5_D0 only, and 12 at both D2_D0 and D5_D0). Those 18 miRNAs were predicted to target 166 mRNAs (Supplemental Table S5), which were enriched for functions related to muscle differentiation and formation (Table 2, Supplemental Table S6).

### 2.8. Chromosome X miRNAs in CMD

Two clusters of five miRNA on chromosome X (miR-450a-5p, miR-503-5p, and miR-542-3p; miR-221 and miR-222) were up- or down-regulated in control mouse and human myoblast differentiation, but not in the DM15 myoblasts (Supplemental Table S7). The mRNA targets of the miR-503 cluster in C2C12 differentiation were enriched for functions related to organismal development and cellular proliferation and differentiation (Figure 4B, Supplemental Table S8). Processes enriched in gene ontology analysis of the miR-221/222 cluster were numerous, including structural morphogenesis, regulation of cell development and differentiation, and regulation of muscle system processes, contraction, and structure development (Supplemental Table S9).

## 3. Discussion

miRNAs are essential regulators of muscle differentiation and understanding their mRNA targets can provide new insight into genetic regulation of development. In this study, we identified miRNAs and mRNAs that are up- or down-regulated during myoblast differentiation to myocytes. Further, we detected differential expression of miRNA that target sarcomere formation and calcium signaling genes during myoblast differentiation. mRNA targets predicted by our data had significant overlap with previously identified targets [25,26,27,28,29,30,31,32,33,34,35], providing support for the bioinformatic approach of requiring both reciprocal expression and miRNA binding sites to identify potential miRNA-mRNA interactions. Expression of genes that were upregulated and predicted to be targeted included the transcription factors *Mef2a*, *Mef2c*, and *MefF2d*, consistent with their role in the expression of sarcomere genes such as *Actc1*, *Acta1*, *Myl1*, and *Tnni1* [24,36]. Known regulators of these transcription factors, miR-92, miR-30c, and miR-10 (Figure 2), were all correctly identified in our studies [25,26,28,29,30]. Our analyses also replicated previous findings of increased myomiR expression during muscle differentiation (miR-1, miR-133a, miR-133b, miR-206, and miR-499) [6,8,37], and correctly predicted their targeting of MEF2 transcription factors and myogenin [31]. Several additional miRNA-mRNA interactions predicted by our methods have also been experimentally validated. These include miR-106b targeting *Mef2d* [32] and *Cdkn1a* [33], miR-322 and miR-503 targeting *Ccnd1* [34], miR-222 targeting *Bmf* [35], and miR-155 targeting *Msi2* [27]. Novel miRNA-mRNA interacts worthy of further investigation include miR-423-5p and *Mef2d*, as miR-423-5p is already known to interact with *Sufu*, a myogenic regulator [26,38].

### 3.1. Calcium Signaling and Sarcomere Function

While miRNA regulation of calcium signaling has been previously described in cardiomyocytes and smooth muscle cells [39,40,41], this is the first identification of miRNAs targeting calcium signaling pathways during skeletal muscle differentiation. The miRNA and target mRNAs predicted by us are unique and different than those characterized in cardiac or smooth muscle [42], reflecting the specific calcium regulation pathways in each muscle type. The four miRNAs targeting calcium channels (miR-221-5p, miR-30c-2-3p, miR-423-3p and miR-423-5p) were downregulated during differentiation, which would allow for increased calcium channel abundance in mature muscle. The majority of the calcium channels predicted to be targeted by miRNA (*Chrnd*, *Chrna1*, *Cacnb1*, and *Cacng6*) have previously been shown to be upregulated during myoblast differentiation [43].

Downstream of calcium channels, calcium-dependent kinases contribute to regulation of sarcomere formation. Many miRNAs were predicted to regulate both calcium signaling and sarcomere gene expression including miR-30c-2-3p (targeting *Cacnb1* in calcium signaling and *Tnni1* in sarcomere formation), miR-92a/b (*Camk2a* and *Actc1*), and miR-423-5p (*Chrnd* and *Actc1*). This integration of calcium signaling and sarcomere function is well characterized, including regulation of muscle-specific MEF2 transcription factors by calcium/calmodulin dependent kinases such as *Camk2* [44]. *Camk2a*, a subunit of CAMK2, is a known target of miR-289 in fruit fly [45]. While the miR-289 family is not present in humans or mice, we predicted several mammalian miRNAs to inhibit *Camk2a* expression in myoblasts including miR-32, miR-92a/b, miR-221, and miR-423-3p. In addition to regulation of calcium signaling, *Camk2a* contributes to the regulation of myosin heavy chain [46,47], slow muscle fiber [14,48,49], and mitochondrial gene [50,51,52] expression in skeletal muscle [53]. This highlights the potential role of these miRNAs to act as indirect regulators of myogenic gene expression through their regulation of *Camk2a.* CAMK2 has been implicated in disorders of both cardiac and skeletal muscle such as cardiac ischemia [54], arrhythmias [55], and limb girdle muscular dystrophy [56]. To date, treatment options have primarily focused on the development of small molecules to inhibit and modulate activity of CAMK2 [57]. Increasing evidence that *Camk2a* is regulated by miRNA suggests miRNAs as potential therapeutic targets in muscle disorders.

### 3.2. miRNA Dysregulation in CMD

The role of miRNAs in muscle disorders was supported by observation that global depletion of miRNAs through *Dicer* knockout in mice lead to impaired muscle development [58]. CMD patient muscle has lower expression of myomiRs such as miR-1 [59], possibly due to differences in miRNA processing [59,60]. In our analysis, we identify 62 miRNA that are differentially expressed during healthy human myoblast differentiation that are not up- or downregulated in differentiating CMD myoblasts. Using the more complete data from the mouse myoblast differentiation, we predicted potential functional consequences of this miRNA dysregulation in CMD. Three miRNAs, miR-155, miR-221, and miR-92b, failed to be downregulated during CMD muscle differentiation, which could lead to inappropriate suppression of the calcium signaling and sarcomere development. Calcium signaling has been shown to be altered in CMD cardiomyocytes, and several genes involved in calcium signaling are differentially expressed in CMD myoblasts [61]. Notably, upregulation of all myomiRs was conserved, supporting the essential role of these miRNAs in muscle development. Additionally, genes known to be implicated in CMD pathogenesis such as *Dmpk* [62], *Rbfox1* [63], and *Cacna1* [59] were differentially expressed during normal C2C12 myoblast differentiation in our data. *Dmpk* was not a predicted target of any miRNA. *Rbfox1* and *Cacna1* were predicted targets of let-7d-3p and miR-423-5p, respectively, but only with moderate confidence. Therefore, they were not considered for further analysis. Furthermore, neither of those two miRNAs were dysregulated during CMD myoblast differentiation. This suggests the study of miRNA could expand our knowledge of the molecular pathways contributing to CMD.

Several additional miRNAs dysregulated in the differentiation of CMD myoblasts are members of miRNA clusters on chromosome X: miR-221/222 and miR-450a/503/542. We observed loss of the expected downregulation of the miR-221/222 cluster and upregulation of the miR-503 cluster during differentiation of CMD myoblasts from a patient with severe disease. The miR-221/222 cluster is known to increase cell proliferation via targeting of p27 tumor suppressor [64] and is a negative regulator of muscle differentiation [65,66]. Over expression of the miR-221/222 cluster inhibits myoblast differentiation [64], so continued expression of this cluster in CMD myoblasts could contribute to abnormal muscle development. Conversely, the miR-503 cluster is a positive regulator of muscle differentiation through inhibition of *Cdc25a*, a kinase responsible for disinhibiting cyclin dependent kinase 2 [67]. During C2C12 myoblast differentiation, miR-503 was predicted to target both *Ccnd1* and *Ccnd2*, which promote proliferation via the activity of cyclin dependent kinases similarly to *Cdc25a*. This is an example of potential de-repression due to lack of miRNA upregulation in CMD. The location of these miRNA clusters on the X chromosome provides a possible mechanism for some of the clinical differences seen between men and women with muscular dystrophies, particularly myotonic dystrophy [68]. More research is needed to determine if there are clinically relevant targets of these clusters.

### 3.3. MyomiRs and Glucuronyltransferases

Here we predict a novel role for miRNAs in regulation of glucuronyltransferase gene expression (including *Ugt1a3*, *Ugt1a4*, *Ugt1a6*, and *Ugt1a8*) during muscle development. In our data, the myomiR miR-486 is predicted to target several UDP-glucuronyltransferases, including *Ugt1a*, which were downregulated during the D2_D0 interval but then upregulated D5_D2. *Ugt1a* encodes UDP-glucuronosyltransferase, an enzyme implicated in skeletal and cardiac muscle differentiation [69], as well as in metabolism of unconjugated bilirubin, thyroxine (T4) and triiodothyronine (T3) [70]. UDP-glucuronosyltransferase is highly expressed in quiescent satellite cells, but is downregulated during differentiation [71]. Previous research has highlighted dysfunction of glucuronyltransferases in CMD. The *LARGE* gene modifies alpha-dystroglycan via glucuronyltransferase activity, depends on cations such as calcium, and is necessary for skeletal muscle sarcomere integrity [72]. Impairment of *LARGE* enzymatic activity has been demonstrated in the mouse model of CMD, and mutations in *LARGE* have been documented in CMD patients [73]. Additional research is needed to further elucidate the role of miRNA, glucuronyltransferases, and calcium signaling in pathogenesis of myotonic dystrophy.

In conclusion, many miRNAs are differentially expressed during muscle differentiation, and predict target mRNAs enrichment for functions related to calcium signaling and sarcomere formation. Limitations to this study include the use of a cell culture model of muscle differentiation, lack of availability of DM13 and DM15 cells to confirm the in silico findings including if C2C12 myotubes at day 5 and human DM13 at day 3 have same level of differentiation, and use of strict criteria for differential expression in prediction of targets. Cell culture models of development differ in many aspects from in vivo development, but nonetheless recapitulate many essential steps of muscle differentiation. It is possible that some miRNAs are targeting mRNAs that do not significantly change in expression and indirect targets would not be detected in our analysis because of the requirement for a miRNA binding site. Important future directions include biochemical confirmation of these predicted regulatory interactions. While some previous computational prediction of miRNA targets has demonstrated poor specificity, requiring anti-correlation of mRNA and miRNA expression increases specificity by providing orthogonal support for regulation [74]. However, the results included here generate new hypotheses about the role of miRNA regulation of calcium signaling and glucuronlytransferase activity in muscle development and disease. Future studies to confirm the many regulatory interactions will be an important next step towards identification of potential therapeutic targets.

## 4. Materials and Methods

### 4.1. Cell Culture

C2C12 mouse myoblasts were cultured and differentiated as per the ENCODE protocol (encodeproject.org, Accessed 26 February, 2015). In brief, myoblasts were grown in DMEM with 20% fetal bovine serum and differentiation initiated by transition to DMEM with 2% horse serum and 1 μM insulin (Gibco, Waltham, MA, USA). Cells were harvested at the specified timepoints and cell pellets were frozen at -80 until RNA extraction.

### 4.2. RNA Extraction and Sequencing

Total RNA was isolated from cell pellets using the RNAEasy kit (Qiagen, Hilden, Germany). RNA and miRNA sequencing libraries were prepared using NEBNext RNA library reagents. Libraries were sequenced on HiSeq 2500 (Illumina, San Diego, CA, USA), 50 bp reads).

### 4.3. mRNA Expression Analysis

mRNA sequencing data were aligned to the mm10 reference genome with TopHat (version 1.4) using the following parameters: ‘--splice-mismatches 1 --min-anchor-length 5 --segment-mismatches 3 --segment-length 25 --max-multihits 0 --no-novel-juncs’, and splice junctions defined by the Illumina iGenome genes.gtf reference. Mitochondrial and duplicate reads were discarded using Samtools and Picard’s MarkDuplicates, respectively. Genes with fewer than 5 counts at 2 or more time points were considered to have low counts and were excluded from the analysis. Differentially expressed genes were identified using the edgeR package for R (version 3.6.0, R Foundation for Statistical Computing, Vienna, Austria). First, effective library sizes were determined using trimmed mean of m values normalization. A common negative binomial dispersion parameter was estimated for the dataset since the samples were run without biological replicates. Gene-specific dispersions were then estimated with the quantile-adjusted conditional maximum likelihood method. Log fold change differential expression was calculated pairwise between each of the three time points (Day 2 vs. Day 0, Day 5 vs. Day 2, and Day 5 vs. Day 0) for both mRNA and miRNA expression using a negative-binomial exact gene-wise test. Significant differential expression was defined as an FDR q-value less than 0.05 and absolute log fold change greater than 1.2 for miRNA and 2.0 for mRNA. Overlap in differentially expressed miRNAs and mRNAs between the D2_D0 and D5_D2 periods were assessed using a hypergeometric distribution, for a total of 253 miRNA and 10287 mRNAs included in analyses after quality filtration.

### 4.4. miRNA-mRNA Correlation Analysis

Predicted miRNA targets were determined using the Ingenuity Pathway Analysis (IPA, Qiagen, Hilden, Germany) miRNA Target Filter feature. IPA predicts miRNA-mRNA interactions at 3 confidence levels: experimentally observed, high confidence, and moderate confidence. Pairings for DE miRNA and mRNAs were identified for each developmental interval (Day 2 vs. Day 0, Day 5 vs. Day 0, and Day 5 vs. Day 2) and filtered for inverse expression (miRNA upregulated when target mRNA downregulated or miRNA downregulated when mRNA target upregulated).

### 4.5. Functional Enrichment

mRNAs that were predicted targets of miRNAs based on experimental observation or a high confidence were assessed for gene ontology enrichment and protein-protein interactions using IPA and ShinyGO v0.60 (bioinformatics.sdstate.edu/go/, Accessed 19 July, 2019). mRNA targeted by myomiRs (miR-1, miR-133a, miR-133b, miR-206, miR-208a, miR-208b, miR-499a, and miR-499b) at all three confidence levels were also analyzed for functional enrichment. Enriched terms with FDR q-values below the pre-determined cutoff of 1 × 10^−3^ were considered significant. The method by which z-scores and *p*-values for functional enrichment are determined by IPA has been previously described [75]. In brief, a positive z-score indicates an increased number of genes that are upregulated and share a common pathway. The *p*-value represents the probability of these genes being present together by chance.

### 4.6. miRNA Expression during CMD Myoblast Differentiation

Differential miRNA expression was determined during differentiation of myoblasts from two CMD and one non-CMD participant myoblasts [76] as described above. The human CMD cell lines, DM13 and DM15, have 1800 and 3200 trinucleotide repeats at the 3′ UTR region of the Dystrophia Myotonic Protein Kinase (*DMPK*) gene, respectively, and are associated with intermediate and severe phenotypes, respectively. Data were downloaded under accession number GSE127296 (ncbi.nlm.nih.gov, accessed on 22 July 2019). The authors report that the DM13 cell line had differentiation comparable to that of the mature myocytes seen in the control cell line at day 3 of differentiation. However, the differentiation of the DM15 cell line was significantly impaired at day 3 [76]. These results are consistent with previous study of impaired differentiation in CMD myoblasts [77]. miRNAs that were differentially expressed in unaffected human myoblasts cells but not in CMD cells were identified, and those that were also found in the mouse genome and expressed in C2C12 cells were assessed for mRNA targets at all three confidence levels (experimentally observed, high confidence, and moderate confidence) using C2C12 paired mRNA-miRNA data. Functional enrichment among those mRNA targets was determined as above.

## Figures and Tables

**Table 1:** Top functional enrichments among differentially expressed mRNAs that are predicted targets of miRNAs. Only the most significantly enriched term among closely related terms was included.

Pathway	D2_D0		D5_D0		D5_D2	
	*p*-Value	z-Score	*p*-Value	z-Score	*p*-Value	z-Score
Calcium Signaling	5.6 × 10^−8^	2.65	1.9 × 10^−6^	2.236	-	-
Estrogen-mediated S-phase Entry	1.3 × 10^−5^	−1.00	3.7 × 10^−4^	-	-	-
Nicotine Degradation	2.3 × 10^−5^	−1.34	-	-	2.6 × 10^−8^	2.00
Melatonin Degradation	3.2 × 10^−5^	−1.34	-	-	3.4 × 10^−8^	2.00
Cell Cycle: G1/S Checkpoint Regulation	5.6 × 10^−5^	1.00	4.8 × 10^−4^	-	-	-
Thyroid Hormone Metabolism	8.3 × 10^−5^	−2.00	-	-	5.2 × 10^−9^	2.00
nNOS Signaling in Skeletal Muscle Cells	1.1 × 10^−4^	-	-	-	-	-
Cyclins and Cell Cycle Regulation	1.4 × 10^−4^	−1.34	9.8 × 10^−4^	-1	-	-
Clathrin-mediated Endocytosis Signaling	-	-	9.1 × 10^−5^	-	-	-
Actin Cytoskeleton Signaling	-	-	2.0 × 10^−4^	-	-	-
PPAR Signaling	-	-	2.6 × 10^−4^	−0.45	-	-
Protein Kinase A Signaling	-	-	3.2 × 10^−4^	1.63	-	-
RhoA Signaling	-	-	5.6 × 10^−4^	1.34		-
Serotonin Degradation	-	-	-	-	5.4 × 10^−8^	2.00
Xenobiotic Metabolism Signaling	-	-	-	-	1.8 × 10^−5^	-

**Table 2:** Top functional enrichments among predicted targets of miRNAs differentially expressed during control human myoblast differentiation but not in CMD myoblasts.

Functional Category	*p*-Value	mRNAs Targets
Muscle structure development	7.4 × 10^−13^	30
Muscle system process	1.3 × 10^−12^	25
Circulatory system development	3.2 × 10^−11^	35
Muscle contraction	6.7 × 10^−11^	21
Anatomical structure formation involved in morphogenesis	1.9 × 10^−10^	35
Muscle organ development	5.1 × 10^−9^	20
Heart development	5.8 × 10^−9^	23
Striated muscle tissue development	1.4 × 10^−8^	19
Response to nitrogen compound	1.9 × 10^−8^	31
Cell differentiation	1.9 × 10^−8^	68
Cellular developmental process	1.9 × 10^−8^	70
Muscle tissue development	1.9 × 10^−8^	19

## Data Availability

Genome-wide differential expression values are contained within the supplementary material.

## Supplementary Materials

Supplementary materials can be found at https://www.mdpi.com/1422-0067/22/5/2692/s1. Table S1: Differential expression of miRNA and mRNAs during C2C12 myoblast differentiation. Table S2: Predicted miRNA targets during C2C12 myoblast differentiation. Table S3: Predicted myomiR targets during C2C12 myoblast differentiation. Table S4: Differential expression of miRNA during human control and CMD myoblast differentiation. Table S5: Predicted targets of miRNA dysregulated in CMD. Table S6: Functional enrichment of predicted targets of miRNA dysregulated in CMD. Table S7: Chromosome X miRNA clusters dysregulated in CMD. Table S8: Predicted targets of the miR-503 cluster. Table S9: Predicted targets of the miR-221/222 cluster.

## Abbreviations

CMD	congenital myotonic dystrophy
mRNA	messenger RNA
miRNA	microRNA
